# Gesundheitskompetente Schule: Konzeptentwicklung für organisationale Gesundheitskompetenz in der Schule

**DOI:** 10.1007/s00103-022-03546-7

**Published:** 2022-05-30

**Authors:** Sandra Kirchhoff, Orkan Okan

**Affiliations:** 1 Interdisziplinäres Zentrum für Gesundheitskompetenzforschung (IZGK), Fakultät für Erziehungswissenschaft, Universität Bielefeld, Bielefeld, Deutschland; 2 Fakultät für Sport- und Gesundheitswissenschaften, Technische Universität München, München, Deutschland; 3 Interdisziplinäres Zentrum für Gesundheitskompetenzforschung (IZGK), Universität Bielefeld, Konsequenz 41a, 33615 Bielefeld, Deutschland

**Keywords:** Gesundheit, Schule, Schulentwicklung, Organisationsentwicklung, Gesundheitsförderung, Health, School, School development, Organization development, Health promotion

## Abstract

**Hintergrund:**

Gesundheitskompetenz gilt heute als wichtige Ressource. Schulen sind bedeutsame Wirkstätten im Leben junger Menschen und können maßgeblich zur Stärkung von Gesundheitskompetenz beitragen. Dies auf dem klassischen verhaltensorientierten Weg über Lernangebote, aber auch über einen verhältnisorientierten Ansatz, indem die Schulorganisation *gesundheitskompetent* optimiert wird. Der Ansatz geht zurück auf das Konzept der organisationalen Gesundheitskompetenz, das bereits in verschiedenen Settings angekommen ist und dabei hilft, Organisationsbedingungen so zu gestalten, dass die Gesundheitskompetenz der jeweiligen Klientel gestärkt wird. Das Projekt GeKoOrg-Schule (Gesundheitskompetente Organisation Schule) folgt diesem Ansatz und überträgt ihn auf das Schulsetting.

**Ziel der Arbeit:**

In GeKoOrg-Schule sollte ein Konzept erarbeitet werden, das Standards zur Entwicklung der organisationalen Gesundheitskompetenz in Schulen bereitstellt und beschreibt, welche Aspekte dabei adressiert werden müssen.

**Material und Methoden:**

Für die Konzeptentwicklung wurde auf bestehende Konzepte zur organisationalen Gesundheitskompetenz zurückgegriffen. Die Ausarbeitung erfolgte mithilfe von Kommentierungs- und Revisionsschleifen, in welche schulische Akteur:innen einbezogen wurden.

**Ergebnisse:**

Das GeKoOrg-Schule-Konzept umfasst acht Standards, die verschiedene Bereiche innerhalb der Schulorganisation zur Optimierung und damit zur nachhaltigen Stärkung von Gesundheitskompetenz aufzeigen.

**Diskussion:**

Die Stärkung von Gesundheitskompetenz in und durch Schule bedarf eines umfassenden verhältnisorientierten Ansatzes. Das Organisationsentwicklungskonzept GeKoOrg-Schule ermöglicht Schulen eine settingbasierte Weiterentwicklung zu gesundheitskompetenten Schulen.

## Abstract (en)

**Background:**

Health literacy is considered a crucial resource nowadays. Schools are important venues for youth that can significantly contribute to promoting health literacy. This can be done in the traditional behavior-focused approach by providing learning opportunities and through an environment-orientated approach by optimizing the school to be a health-literate organization. This perspective is based on the concept of organizational health literacy, which is used in different settings to change organizational conditions to strengthen the health literacy of the respective clientele. The project “HeLit-Schools” (Health-Literate Schools) follows this approach and transfers it to the school setting.

**Objectives:**

In the HeLit-Schools project, a concept should be developed that provides standards for the development of organizational health literacy in schools and identifies which aspects need to be addressed.

**Materials and methods:**

The concept development was based on existing concepts of organizational health literacy. The concept was developed by involving key school stakeholders in commenting and revision loops.

**Results:**

The HeLit-Schools concept includes eight standards that depict different areas within the school organization for optimizing and thus sustainably strengthening health literacy.

**Conclusions:**

Promoting health literacy in and through schools requires a comprehensive environment-oriented approach. The organization development concept HeLit-Schools enables schools to become health-literate using setting-based development.

## Hintergrund

Gesundheitskompetenz lässt sich als maßgebliche und unterstützende Fähigkeit für den Umgang mit Gesundheitsinformationen und -wissen sowie als Ergebnis von Bildungs- und Erziehungsprozessen fassen [1]. Als bedeutsame individuelle Ressource wird Gesundheitskompetenz daher in den letzten Jahren in Deutschland vermehrt als wichtiges Bildungsziel beschrieben und taucht prominent in Empfehlungen sowohl des Nationalen Aktionsplans Gesundheitskompetenz [2] als auch in der Allianz Gesundheitskompetenz und Schule [3] auf.

Die Relevanz von Gesundheitskompetenz nimmt stetig zu, insbesondere durch gegenwärtige gesellschaftliche Entwicklungen und Herausforderungen, wie die fortschreitende Digitalisierung verschiedener Lebenswelten [4]. Gesundheitsbezogene Informationen sind mittlerweile durch digitale und soziale Medien jederzeit und überall verfügbar. Auch lässt sich im Mediennutzungsverhalten junger Menschen die zunehmende Bedeutung dieser Informationsquellen ablesen [5, 6]. Darüber hinaus zeigt auch die Coronapandemie die Notwendigkeit von Kompetenzen für den Umgang mit Gesundheitsinformationen. Zwar stellte die Informationsflut (*Information Overload*) bereits vor der Pandemie eine große Herausforderung dar [7], diese wurde jedoch durch die Pandemie und die einhergehende *Infodemie*^1^ immens verschärft [9].

Diese exemplarischen Entwicklungen sprechen dafür, Gesundheitskompetenz verstärkt als wichtiges Bildungsziel anzuerkennen und möglichst früh im Leben mit kompetenzfördernden Maßnahmen anzusetzen [10]. Bildungseinrichtungen wie Schulen kann hier eine Schlüsselrolle in der Vermittlung von Gesundheitskompetenz zukommen. Durch Bildungsangebote können Schüler:innen früh die Grundlagen erlernen, aus denen sich im weiteren Lebensverlauf eine hohe Gesundheitskompetenz entwickeln lässt [11]. Darüber hinaus besteht eine indirekte Verbindung zwischen Bildungsergebnissen und Gesundheitskompetenz: Während Gesundheitskompetenz direkten Einfluss auf das Gesundheitsverhalten und den Gesundheitszustand hat, wirken sich besagte gesundheitliche Outcomes positiv auf Bildungsergebnisse aus, da Gesundheit und Bildung sich wechselseitig beeinflussen [12]. Außerdem kann die gesundheitliche Chancengleichheit durch Gesundheitskompetenz erhöht werden, da Gesundheitskompetenz als eine Determinante von Gesundheit gilt, die explizit durch unterschiedliche Fördermaßnahmen verändert werden kann [13–15].

Neben der direkten Adressierung der individuellen Gesundheitskompetenz junger Menschen in der Schule kann auch an der Schulorganisation angesetzt und eine sogenannte organisationale Gesundheitskompetenz etabliert werden. Dies dient dazu, über rein verhaltensorientierte Maßnahmen hinweg auch die *Verhältnisse* in den Blick zu nehmen. Damit sind Strukturen und Bedingungen der schulischen Lebenswelt gemeint, die oft größeren Einfluss auf Kompetenz, Verhalten und Gesundheit haben [12, 16].

### Organisationale Gesundheitskompetenz

Bei der Gesundheitskompetenz wirken persönliche Fähigkeiten und Kapazitäten mit Anforderungen und Komplexitäten der Umwelt zusammen [17]. Gesundheitskompetenz und gesundheitskompetentes Handeln hängen also maßgeblich davon ab, inwieweit die individuellen Fähigkeiten sowie die Komplexität, Verständlichkeit und Zugänglichkeit von gesundheitsbezogenen Informationen, Angeboten und Systemen (im weitesten Sinne die „Umweltbedingungen“) miteinander harmonieren. Demnach müssen bei der Beschäftigung mit Gesundheitskompetenz stets die Einbindung in die jeweilige Umwelt und deren Beschaffenheit mitbetrachtet werden.

Diese Dualität von Gesundheitskompetenz wird im relationalen Modell nach Parker und Ratzan (2010; [17]) beschrieben und wurde jüngst durch Sørensen et al. (2019; [18]) mit der Umkehrung des Modells ergänzt. Hiernach gilt es ebenso, die Kompetenzen in einem System (z. B. des Personals/der Einrichtung) im Zusammenspiel mit den Komplexitäten der Individuen, die das jeweilige System nutzen, zu berücksichtigen.

Zur Stärkung von Gesundheitskompetenz lassen sich daraus zwei Ansatzpunkte bestimmen: Erstens können unterschiedliche (Bildungs‑)Maßnahmen direkt auf individuelle Fähigkeiten abzielen (verhaltensorientierter Ansatz). Zweitens kann an der Zugänglichkeit und Verständlichkeit von gesundheitsrelevanten Informationen, Angeboten und Systemen [19] bzw. der sogenannten Responsivität von Organisationen [20] angesetzt werden (verhältnisorientierter Ansatz). Denn auch Umweltbedingungen können *gesundheitskompetent* gestaltet sein, sodass sie den Umgang mit Gesundheitsinformationen erleichtern, individuelle Gesundheitskompetenz stärken und die Ausbildung von gesundheitsförderlichen Einstellungen und Verhaltensweisen fördern [17].

Die Perspektive des relationalen Modells von Gesundheitskompetenz wird im Konzept der *gesundheitskompetenten Organisation* aufgegriffen (Infobox 1). Dieses Konzept beinhaltet einen ganzheitlichen Ansatz, der auf Fördermaßnahmen zur Stärkung der individuellen Gesundheitskompetenz auf der Verhaltensebene und auf Maßnahmen zur Ausrichtung und Optimierung von organisationalen Bedingungen und Strukturen auf der Verhältnisebene setzt.

Das Konzept geht zurück auf Arbeiten in den USA zur Verbesserung der Gesundheitsförderung und Krankenversorgung. Neben der Stärkung der individuellen Gesundheitskompetenz sollten Organisationen im Versorgungssystem gesundheitskompetent gestaltet werden, sodass Organisationsstrukturen den Bedarfen der Nutzer:innen entsprechen (Zugänglichkeit, Transparenz etc.), um Behandlungsergebnisse und Kommunikation zwischen Fachkräften und Nutzer:innen zu verbessern sowie individuelles Gesundheitsverhalten positiv zu beeinflussen [21].

Basierend auf Vorarbeiten wurde 2012 ein integrierter Ansatz organisationaler Gesundheitskompetenz für Organisationen im Versorgungssystem vom US-amerikanischen Institute of Medicine (IOM)^2^ veröffentlicht [23]. Die dort formulierten zehn Merkmale gesundheitskompetenter Organisationen haben Pelikan und Dietscher (2015) zum Wiener Konzept Gesundheitskompetenter Krankenbehandlungsorganisationen (WKGKKO) weiterentwickelt, in dem neben der Gesundheitskompetenz von Patient:innen auch die von Mitarbeiter:innen und Akteur:innen im Gesundheitssektor adressiert und die Unterstützung der regionalen Umwelt betont wird [19].^3^

Der Ansatz der organisationalen Gesundheitskompetenz findet seit Kurzem auch Berücksichtigung in anderen Settings, z. B. in der sozialen Arbeit, innerhalb ganzheitlicher Strategien zur Gesundheitsförderung und Prävention. 2016 wurde in Österreich eine Adaption des WKGKKO für den Kontext der offenen Jugendarbeit veröffentlicht und in der Praxis angewendet [26]. Für Schulen wird an und mit ähnlichen Konzepten in den USA [27], in Australien [20, 28, 29] sowie jüngst auch in Österreich [30] gearbeitet. Diese Anwendungsbeispiele zeigen die Anschlussfähigkeit des Konzepts im Bildungssetting. Die Passung beruht zum einen darauf, dass Gesundheitskompetenz als Bindeglied zwischen Gesundheit und Bildung verstanden werden kann [31]. Zum anderen lassen sich diverse Anschlussstellen von Gesundheitskompetenz zu Schulkonzepten der Gesundheitsförderung, z. B. die *gute gesunde Schule* [32], oder Empfehlungen der Kultusministerkonferenz (KMK; [33]) herausarbeiten.

Da Schulen aus System- und Organisationsperspektive als Organisationen gelten [34], ist der Ansatz der gesundheitskompetenten Organisation übertragbar. Schulen sind dann gesundheitskompetente Organisationen, wenn die Ausrichtung der Schulorganisation, -struktur und der Rahmenbedingungen dazu beiträgt, dass alle Personengruppen in der Schule individuelle Gesundheitskompetenz entwickeln, einüben und stärken können [16]. Vorarbeiten von Pelikan (2017, 2019; [21, 35]) sowie Pelikan und Dietscher (2015; [19]) legen nahe, dass ganzheitliches Ansetzen und Organisationsoptimierung als umfassender und damit nachhaltiger und kosteneffizienter einzuschätzen sind als Vorhaben, die ausschließlich die individuelle Gesundheitskompetenz stärken. Die anvisierten positiven Veränderungen bauen so nicht nur auf den vorhandenen bzw. erstellten Lernangeboten, der Lernmotivation und/oder den Fähigkeiten der Schüler:innen auf – sie werden maßgeblich von Schulorganisation, Schulumfeld und zentralen Akteur:innen unterstützt und umgesetzt.

Vor diesem Hintergrund soll ein Organisationsentwicklungskonzept entwickelt werden, das Standards zur Entwicklung der organisationalen Gesundheitskompetenz in Schulen bereitstellt und die zu adressierenden Aspekte beschreibt: Was charakterisiert eine gesundheitskompetente Schule? Welche inhaltlichen Aspekte und schulorganisatorischen Ebenen müssen in einem ganzheitlichen Organisationsansatz adressiert werden, um eine Schule zu einer gesundheitskompetenten Organisation zu entwickeln? Nachfolgend werden die Projekteinbettung und Vorgehensweise der Konzeptentwicklung, die auf der Adaption bestehender Konzepte organisationaler Gesundheitskompetenz basiert, beschrieben. Daran anschließend werden die im Konzept erarbeiteten Standards der gesundheitskompetenten Schule präsentiert und aufgezeigt, wie diese im Praxisfeld Schule Anwendung finden können.

## Methoden

### Das Projekt GeKoOrg-Schule

Das Projekt GeKoOrg-Schule^4^ zielt darauf ab, ein deutschsprachiges Konzept organisationaler Gesundheitskompetenz für Schulen zu entwickeln. Gemäß umfassender und verhältnisorientierter Ausrichtung wird im Projekt das Konzept der gesundheitskompetenten Organisation auf das Setting Schule übertragen [16, 36, 37]. Das Konzept bezieht sich demnach auf einen Ansatz schulbasierter Organisationsentwicklung, wobei Organisationsentwicklung als prozessorientierter, ganzheitlicher Ansatz zu fassen ist, der durch hohe systemische Prägung sowie langfristig ausgerichtete Planung und kontinuierliche Weiterentwicklung der Organisation Schule mittels gesteuerter Veränderungsprozesse charakterisiert wird [34]. Entwicklung durch Veränderung wird hier explizit aus dem Inneren der Organisation herbeigeführt. Die Organisationsmitglieder sind die zentralen Akteur:innen für die Realisierung und Ausgestaltung dieser Entwicklungs- und Veränderungsprozesse. Über diesen Ansatz sollen schulische Strukturen, Rahmenbedingungen, Netzwerke und Kooperationen in Bezug auf Gesundheitskompetenz erfasst, verändert und justiert werden [36, 37].

Im Projekt werden das Konzept der gesundheitskompetenten Schule (GeKoOrg-Schule-Konzept) inklusive sogenannter Standards einer gesundheitskompetenten Schule, ein Umsetzungsleitfaden und ein Messinstrument zur Erfassung der organisationalen Gesundheitskompetenz von Schulen entwickelt. Zudem ist eine Messung geplant, die schulübergreifend Einblicke in Gelingensbedingungen und Entwicklungsbedarfe auf struktureller und inhaltlicher Ebene innerhalb der Schulorganisation verspricht. Damit können wichtige Anhaltspunkte für die systemische Stärkung der Gesundheitskompetenz im Schulalltag gewonnen werden.

Um Synergien zu nutzen und die praktische Umsetzung des Konzepts zu fördern, soll im Projektverlauf das Thema Gesundheitskompetenz an bestehende und erfolgreiche Konzepte schulischer Gesundheitsförderung angeschlossen werden, um an gemachten Erfahrungen anzuknüpfen und zugleich Passung und Anschlussfähigkeit herauszustellen. Konkret soll die Verknüpfung zwischen dem GeKoOrg-Schule-Konzept und dem Ansatz der *guten gesunden Schule* [32] herausgearbeitet werden. Ein weiterer Eckpfeiler ist die Gründung der „Allianz Gesundheitskompetenz und Schule“, die in der Entwicklung, Umsetzung und Evaluation von Maßnahmen schulischer Gesundheitskompetenzförderung aktiv werden und zur Nachhaltigkeit beitragen soll [3].

### Konzeptentwicklung und -adaption

Die Konzeptentwicklung stützte sich im Kern auf Adaptions- und Anpassungsarbeiten vorhandener Konzepte organisationaler Gesundheitskompetenz in anderen Settings. Als zentrale Bezugsquellen wurden das WKGKKO [19] und das Konzept der *Gesundheitskompetenten Offenen Jugendarbeit* des österreichischen bundesweiten Netzwerks für Offene Jugendarbeit (bOJA) sowie des Bundesnetzwerks Österreichische Jugendinfos (BÖJI) in den Adaptionsprozess einbezogen [26]. Das bOJA/BÖJI-Konzept basiert auf dem WKGKKO und ist der Übertrag auf das Setting der offenen Jugendarbeit, das mit Jugendlichen bereits eine für Schule relevante Zielgruppe ins Handlungszentrum stellt. Dieses Konzept ist passfähig und übertragbar auf das GeKoOrg-Schule-Vorhaben, da Zielgruppe und Tätigkeitsbereiche der offenen Jugendarbeit denen der Schule ähneln und Bildungsinhalte und -aufträge vergleichbar sind. Darüber hinaus gibt die berichtete Vorgehensweise des Konzepttransfers wertvolle Hinweise für die Entwicklung des GeKoOrg-Schule-Konzepts.

Geplant war, die Konzeptarbeiten mit partizipativen Workshops mit Expert:innen aus dem Schul- und Bildungsbereich zu begleiten. Dies konnte aufgrund der Coronaviruspandemie nicht umgesetzt werden. Um dennoch die Expertise relevanter Akteur:innen einzuschließen, beteiligten sich Expert:innen aus der Schulpraxis, dem Gesundheitssektor sowie der Gesundheitskompetenzforschung in wiederholten Kommentierungs- und Revisionsschleifen auf digitalem Weg an der Konzeptentwicklung. Nachdem eine erste Konzeptversion vom Projektteam erarbeitet worden war, prüften und kommentierten ein wissenschaftlicher Projektbeirat (sechs Personen mit Expertise in den Forschungsbereichen Gesundheitskompetenz, schulische Gesundheitsförderung und Prävention sowie Gender und Diversity) sowie Partner:innen aus Ministerien, Wissenschaft und Praxis das Konzept. Jede Rückmeldung wurde von zwei Projektmitarbeitenden unabhängig voneinander begutachtet und eingeschätzt. Im zweiten Schritt wurden die Einschätzungen und resultierenden Veränderungsvorschläge im Projektteam diskutiert und bei begründetem Konsens ins Konzept eingearbeitet. Eine Validierungsschleife wurde intern am Interdisziplinären Zentrum für Gesundheitskompetenzforschung (IZGK) vorgenommen. Die fortgeschrittene Konzeptversion wurde dann zur Überprüfung Lehrkräften und Schulleitungen in Bielefeld vorlegt, die über Praxispartner:innen angesprochen und bei Kommentierungsbereitschaft vom Projektteam kontaktiert wurden.

Die Rückmeldungen von Beirat und Partner:innen bezogen sich auf Konzeption und Formulierung der Standards und Indikatoren (z. B. Reduktion und Zusammenführung von Standards, Überarbeitung von Indikatoren, Auflösung von Subindikatoren). Die Kommentare aus der Bildungspraxis betrafen hingegen konkrete bildungs- und schulbezogene Inhalte und Arbeitsweisen. Es wurde auf Organisationsprozesse, Aufgaben von Schulaufsicht und -träger sowie Interaktionspunkte mit dem nahen Schulumfeld und Eltern hingewiesen. Die hier genannten Aspekte wurden im ursprünglichen Modell nicht berücksichtigt oder waren verstärkt auf die soziale und Gemeindearbeit und Jugendzentren ausgerichtet. Zudem wurden Verschiebungen von Indikatoren zwischen den Standards empfohlen, da bestimmte Aspekte z. B. in den Aufgabenbereich der Schulleitung fallen (Entwicklung und Definition des schulischen Leitbilds, Unterstützung/Genehmigung von gesundheitsbezogenen Maßnahmen, Kommunikation mit Stadt und Behörden) und andere strukturell zur Dimension der Schulentwicklung zählen (Zeit‑, Arbeits- und Ressourcenplanung, Schulentwicklungspläne, außercurriculare Aktivitäten, Fortbildungen). Weiterhin wurden Konkretisierungen vorgeschlagen, z. B. bzgl. Aktivitäten und Angebote für Eltern (wie Elternabende, Projekttage). Auch gingen Hinweise ein zu Fortbildungsangeboten für Lehrkräfte, zur praxisgerechten Abbildung von Funktionen in der Schule, zur Verknüpfung von Schulinhalten und Themen der Gesundheitsförderung und Prävention, die bereits im Schulalltag verankert sind, sowie zu unrealistischen Anforderungen des Konzepts bzgl. Aufgaben zur Stärkung der Gesundheitskompetenz.

In der finalen Phase der Konzeptarbeiten wurde der Leitfaden *Schulen für Gesundheitskompetenz* in Österreich von der Non-Profit-Organisation Styria vitalis veröffentlicht [30]. Die Arbeiten von Styria vitalis beziehen sich ebenfalls auf Vorarbeiten zur organisationalen Gesundheitskompetenz in Österreich. Die Berücksichtigung dieses Leitfadens in der Konzeptfinalisierung trug insbesondere zur Vereinfachung des GeKoOrg-Schule-Konzepts und zur Reduzierung von Indikatoren bei.

## Ergebnisse

Insgesamt umfasst das GeKoOrg-Schule-Konzept acht Standards, die als Leitlinien zu verstehen sind. Diese beschreiben unterschiedliche Bereiche bzw. Ebenen innerhalb der Schulorganisation, die zur Optimierung adressiert werden, um eine nachhaltige Stärkung der Gesundheitskompetenz im Setting Schule zu erreichen. Schulen sind aus System- und Organisationsperspektive komplexe, soziale Systeme [34], die maßgeblichen Einfluss auf Personen nehmen können, die sich in der Schule befinden – auch beim Thema Gesundheit. Dafür ist es wichtig, bei der Organisationsentwicklung alle relevanten Ebenen zu berücksichtigen, um der Vielschichtigkeit der Schulorganisation gerecht zu werden und das gesundheitsförderliche Potenzial bestmöglich auszuschöpfen [3, 32]. Eine entsprechende Definition der *gesundheitskompetenten Schule* ist Infobox 2 zu entnehmen.

Die Ausrichtung einer Schule zur gesundheitskompetenten Organisation kann im Rahmen von Schulentwicklungsprozessen vollzogen werden. Auch in Schulentwicklungsprozessen gilt es, verschiedene Ebenen der Schulorganisation zu adressieren, da sie sich wechselseitig beeinflussen und so angestrebte Veränderungen umfassend und nachhaltig erzielt werden können. Relevante Ebenen gemäß dem Drei-Wege-Modell nach Rolff (2016) sind Organisation, Unterricht und Personal [34]. Aufbauend wird von Okan et al. (2021; [3]) und Dadaczynski et al. (2021; [38]) auf die notwendige Berücksichtigung einer weiteren Ebene hingewiesen: die des außerschulischen Umfelds. Neben Schulen sind andere Bezugssysteme (z. B. Familie) wichtige und mitgestaltende Bestandteile der Lebenswelt. Entsprechend müssen in einem ganzheitlichen Ansatz diese außerschulischen Wirkfaktoren und -systeme mitgedacht werden. Die Standards des GeKoOrg-Schule-Konzepts berücksichtigen alle vier Ebenen, wodurch die Ansprüche ganzheitlicher Settingansätze erfüllt werden.

### Standards des GeKoOrg-Schule-Konzepts

Einen Überblick über die entwickelten Standards liefert Tab. 1. Im Folgenden werden die acht Standards näher erläutert.

**Table 1:** 

Acht Standards der gesundheitskompetenten Schule (GeKoOrg-Schule-Konzept)		
1	Gesundheitskompetenz in das Leitbild der Schule aufnehmen	Standard 1 sieht vor, dass Schulleitung, Lehrkräfte und Schulpersonal die Stärkung der Gesundheitskompetenz in der Schule als wichtig erachten
2	Gesundheitskompetenz als Teil der Schulentwicklung	Standard 2 verortet Gesundheitskompetenz auf der Organisations- und Schulentwicklungsebene
3	Gesundheitskompetenz im Schulalltag stärken und fördern	Standard 3 stellt sicher, dass die Ausgestaltung des Schulalltags dazu beiträgt, Gesundheitskompetenz an der Schule zu fördern
4	Gesundheitskompetenz für Schüler:innen	Standard 4 widmet sich der konkreten Stärkung der Gesundheitskompetenz von Schüler:innen im Schul- und Unterrichtsalltag
5	Ein gesundheitskompetentes Schulteam	Standard 5 fokussiert die Stärkung der Gesundheitskompetenz von Schulleitungen, Lehrkräften und Schulpersonal
6	Gesundheitskompetente Kommunikation in der Schule	Standard 6 achtet auf einfaches, verständliches Kommunizieren als Prinzip einer gesundheitskompetenten Schule
7	Gesundheitskompetenz im Schulumfeld stärken	Standard 7 setzt Gesundheitskompetenz im Rahmen schulischer Gesundheitsförderung und Prävention für ein gesundes Schulumfeld ein
8	Vernetzen und Zusammenarbeiten	Standard 8 stellt die Beteiligung an Netzwerken, die Kooperation und den Austausch zum Thema Gesundheitskompetenz in den Vordergrund

*Standard 1 *spricht für die Aufnahme von Gesundheitskompetenz in das schulische Leitbild bzw. die Schulphilosophie. Einhergehend gilt es, dass Schulleitungen, Lehrkräfte und weiteres Schulpersonal Gesundheitskompetenz und deren Stärkung in und durch Schule als bedeutsam erachten und als Ziel schulischer Gesundheitsförderung und Prävention anerkennen. Die Umsetzung von Standard 1 profitiert durch die Unterstützung seitens der Schulleitung einerseits, da diese eine Schlüsselfunktion in Veränderungsprozessen der Schule innehat. Andererseits ist auch die Zuwendung durch die Schulträger in Form von Ressourcen zuträglich.

*Standard 2* schließt daran an und positioniert das Thema Gesundheitskompetenz auf der Schulentwicklungsebene. Es sollten Ansprechpersonen in der Schule für das Thema benannt, Maßnahmen zur Stärkung der Gesundheitskompetenz im und außerhalb des Unterrichts geplant, umgesetzt und weiterentwickelt werden sowie personelle, zeitliche und finanzielle Ressourcen zur Verfügung stehen.

*Standard 3* widmet sich der Ausgestaltung des Schulalltages, die dazu beitragen soll, Schüler:innen darin zu unterstützen, Gesundheitskompetenz zu entwickeln und zu erproben. Die Ausgestaltung beinhaltet unterschiedliche Maßnahmen, wie beispielsweise die Anpassung bestehender Unterrichtsinhalte und -methoden, zugängliche Informationsangebote zur Gesundheit, die lebensweltnah, altersgerecht und diversitätssensibel sind, oder gesundheitskompetentes Vorbildhandeln des Schulteams.

*Standard 4 und 5* knüpfen hier an und fokussieren die Stärkung der individuellen Gesundheitskompetenz unterschiedlicher Adressat:innen in der Schule: *Standard 4* richtet sich an die Schüler:innen. Zur Stärkung ihrer individuellen Gesundheitskompetenz sollen im Schul- und Unterrichtsalltag Lernangebote geschaffen werden: Es soll Unterrichtsmaterialien und -inhalte geben, mithilfe derer sich Schüler:innen Gesundheitswissen und Kompetenzen erarbeiten können, um Gesundheitsinformation unterschiedlicher Quellen verstehen, kritisch hinterfragen und anwenden zu können. Dabei ist bei der Auswahl und Gestaltung der gesundheitsbezogenen Inhalte darauf zu achten, dass diese den Interessen, Wünschen und Bedarfen der Schüler:innen entsprechen. Das Thema Gesundheitskompetenz lässt sich an unterschiedlichen Stellen im Rahmen der schulischen Gesundheitsförderung und Prävention und als Querschnittsthema z. B. im Rahmen der digitalen Bildung oder Medienbildung aufgreifen. In *Standard 5* wird das Schulteam, bestehend aus Schulleitungen, Lehrkräften und weiterem Schulpersonal, adressiert. Es gilt, Fort- und Weiterbildungsmöglichkeiten bereitzustellen und in Anspruch zu nehmen, die zur Stärkung der individuellen Gesundheitskompetenz beitragen. Zudem ist es ein wichtiges Anliegen, die Gesundheit des Schulteams zu adressieren und durch Maßnahmen und Verbesserungen der schulischen Rahmenbedingungen zu fördern.

*Standard 6* stellt einfache, verständliche Kommunikation als Prinzip gesundheitskompetenter Schulen vor. Gespräche im Schulkontext zu und über gesundheitsbezogene Themen sollten so gestaltet werden, dass sie für alle einfach und verständlich sind (Schüler:innen, Eltern bzw. Erziehungsberechtigte etc.). Es gilt, für die Bedeutsamkeit von Kommunikation über gesundheitliche Themen (körperliche sowie psychische Gesundheit) zu sensibilisieren. Gleichzeitig sollten notwendige Fähigkeiten in Unterricht und Schulalltag vermittelt werden, dazu gehören z. B. das kritische Hinterfragen oder verständnisförderndes Kommunizieren. Da dafür auch Kenntnisse aufseiten der Vermittler:innen (z. B. Lehrkräfte) vonnöten sind, sollten hier Fort- und Weiterbildungsangebote anschließen, die Kommunikationsmethoden zur Vermittlung von gesundheitlichen Themen beinhalten.

*Standard 7* und *8* beziehen sich auf das engere und weitere Schulumfeld: *Standard 7* beschreibt einerseits, dass für ein gesundes Schulumfeld Gesundheitskompetenz in schulische Gesundheitsförderung und Prävention integriert werden sollte und genutzt werden kann, um Ziele schulischer Gesundheitsförderung zu erreichen. Andererseits soll hier auf Kooperation mit schulischen und außerschulischen Unterstützungs- und Gesundheitsdiensten gesetzt werden, damit Schule als (Erst)Anlaufstelle eine vermittelnde Rolle bei gesundheitsrelevanten Anliegen der Schüler:innen einnehmen kann. Auch gilt es, bei schulischen Gesundheitsthemen Eltern bzw. Erziehungsberechtigte als wichtige Bezugspersonen anzuerkennen und zu involvieren. *Standard 8* setzt ebenfalls auf Vernetzung, Kooperation und (Erfahrungs‑)Austausch zum Thema Gesundheitskompetenz, allerdings liegt hier der Fokus explizit auf dem außerschulischen Umfeld und regionalen schul- sowie gesundheitsrelevanten Akteur:innen.

Die kurz umfassten Inhalte und Ziele der Standards werden in je sechs Indikatoren ausformuliert. Diese werden in einem Fragebogen abgebildet, mit dessen Hilfe die organisationale Gesundheitskompetenz gemäß dem GeKoOrg-Schule-Konzept erfasst werden kann [39]. Schulen können den Fragebogen beispielweise im Rahmen von Schulentwicklungsprozessen zur Bestandsaufnahme anwenden. Die mit dem Fragebogen vorgenommene Selbsteinschätzung kann dabei helfen, die Schulorganisation hinsichtlich ihrer *Gesundheitskompetenzfreundlichkeit* zu überprüfen: Welche Strukturen, Rahmenbedingungen, Abläufe usw. im Schulalltag stärken die Gesundheitskompetenz? Auf welcher Ebene bzw. in welchem Bereich befinden sich Leerstellen und zugleich Anknüpfungspunkte für die Planung und Realisierung von Veränderungen und Maßnahmen, um die Schule zu einer gesundheitskompetenten Schule weiterzuentwickeln? Der Fragebogen ist also ein Instrument, das Entwicklungsbereiche und Bezugspunkte innerhalb der Schulorganisation für eine Weiterentwicklung aufzeigen kann.

## Diskussion

Das GeKoOrg-Schule-Konzept beinhaltet einen ganzheitlichen Organisationsentwicklungsansatz zur Stärkung der Gesundheitskompetenz in Schulen, der neben der Adressierung der individuellen Ebene im Sinne der Verhaltensprävention auch explizit die strukturelle Ebene der Schulorganisation und somit die Verhältnisprävention einschließt. Nach unserem Kenntnisstand ist dies das erste umfassende Konzept organisationaler Gesundheitskompetenz für Schulen in Deutschland.

Die Standards berücksichtigen nicht nur die relevanten Ebenen zur Schulentwicklung (Personal, Unterricht, Organisation, Umfeld), sondern lassen sich auch entlang gängiger Handlungsfelder schulischer Gesundheitsförderungsansätze einordnen [12]. Die Bedeutsamkeit der Stärkung individueller Gesundheitskompetenz in und durch Schule wird seit einiger Zeit betont [1, 11, 12, 40, 41]. Neben Schüler:innen sind Schulleitungen und Lehrpersonen wichtige Adressat:innen: Schulleitungen nehmen eine Schlüsselfunktion für schulische Entwicklung und die Ermöglichung von Maßnahmen zur Gesundheitsförderung ein [42, 43], während Lehrkräfte ausschlaggebend für das Lernen und die Ausgestaltung von Interaktionen sind [11]. Das Schulumfeld ist ebenfalls als wichtige Wirkgröße gesetzt, sodass regionale Organisationen, Dienste etc. die Stärkung von Gesundheitskompetenz in der Schule unterstützen [11, 12].

Der Organisationsentwicklungsweg hin zur gesundheitskompetenten Schule ist lohnend, aber voraussetzungsvoll. Das liegt daran, dass der verhältnisorientierte Ansatz die Ausrichtung der gesamten Schulorganisation in den Blick nimmt. Während die aufgezeigten Vorteile im Bereich effektiverer und nachhaltiger Veränderungen und Reichweite liegen, kann die konkrete Umsetzung als herausfordernd eingeschätzt werden, da viele Stellschrauben anzusteuern und auch zeitliche, personale und finanzielle Ressourcen einzubringen sind.

Mithilfe der im Konzept ausformulierten Standards und eines darauf basierenden Fragebogens wird Schulen die Möglichkeit eröffnet, Bedarfe, Potenziale, vorhandene Strukturen und Bedingungen für das Thema Gesundheitskompetenz aus einer Organisationsentwicklungsperspektive systematisch zu erfassen, um Veränderungen und Weiterentwicklungsprozesse zu planen, zu realisieren und zu überprüfen.

Zum aktuellen Zeitpunkt fehlt im Hinblick auf die praktische Umsetzung des Konzepts im Schulfeld die empirische Basis, um detailliert Auskunft über Gelingensbedingungen und Barrieren zu geben. Allerdings steht im GeKoOrg-Schule-Projekt die Messung organisationaler Gesundheitskompetenz von Schulen an, zur empirischen Konzeptvalidierung und zur Identifizierung schulübergreifender, förderlicher sowie hinderlicher Bedingungen, aus denen Umsetzungsempfehlungen abgeleitet werden.

### Infobox 1 Begriffsbestimmung „gesundheitskompetente Organisation“

Eine *gesundheitskompetente Organisation* unterstützt Menschen, die sich in der Organisation aufhalten, bestmöglich dabei, auf Gesundheitsinformationen zuzugreifen, sie zu verstehen, zu bewerten und anzuwenden, um den Alltag gesundheitsfördernd zu gestalten. Gesundheitskompetente Organisationen ermöglichen also, dass die individuelle Gesundheitskompetenz entwickelt und gesundheitskompetentes Handeln gestärkt wird (Übersetzung in Anlehnung an Brach/Harris 2021 [22]).

### Infobox 2 Definition „gesundheitskompetente Schule“

Eine *gesundheitskompetente Schule* gestaltet Prozesse, Strukturen und Rahmenbedingungen so, dass in ihrem Setting Gesundheitskompetenz entwickelt, eingeübt und gefördert werden kann, um alle Personen in der Schule – Schüler:innen, Schulleitungen, Lehrkräfte und nicht unterrichtendes Personal, aber auch Eltern/Erziehungsberechtigte und Personen des erweiterten Schulumfelds – für den Umgang mit Gesundheitsinformationen zu befähigen und gesundheitskompetentes Handeln zu stärken [16].

## Footnotes

1 Infodemie (bzw. Informationsepidemie) beschreibt die stetig steigende Anzahl und schnelle Verbreitung von Informationen – auch Falschinformationen, die die Suche nach vertrauenswürdigen Inhalten und die Bewertung dieser erschweren [8].

2 Institute of Medicine der US-amerikanischen National Academy of Medicine.

3 Weitere Arbeiten zur organisationalen Gesundheitskompetenz werden z. B. von Meggetto et al. (2018; [24]) oder Farmanova et al. (2018; [25]) systematisiert.

4 GeKoOrg-Schule steht für „Gesundheitskompetente Organisation Schule“ und ist der Titel eines vom Bundesministerium für Gesundheit geförderten Projekts, das am Interdisziplinären Zentrum für Gesundheitskompetenzforschung (IZGK) der Universität Bielefeld und an der Technischen Universität München durchgeführt wird. Weiterführende Informationen unter: www.gekoorg-schule.de.
